# Unlocking Cowpea’s Defense Responses: Conserved Transcriptional Signatures in the Battle against CABMV and CPSMV Viruses

**DOI:** 10.3390/life13081747

**Published:** 2023-08-15

**Authors:** Artemisa Nazaré Costa Borges-Martins, José Ribamar Costa Ferreira-Neto, Manassés Daniel da Silva, David Anderson de Lima Morais, Valesca Pandolfi, Roberta Lane de Oliveira Silva, Ana Luiza Trajano Mangueira de Melo, Antônio Félix da Costa, Ana Maria Benko-Iseppon

**Affiliations:** 1 Departamento de Ensino, Instituto Federal do Maranhão, Buriticupu 65393-000, Brazil; artemisa.borges@ifma.edu.br; 2 Departamento de Genética, Centro de Biociências, Universidade Federal de Pernambuco, Recife 50670-901, Brazil; manasses.dsilva@ufpe.br (M.D.d.S.); valesca.pandolfi@ufpe.br (V.P.); analuiza.melo@ufpe.br (A.L.T.M.d.M.); 3 Centre de Recherche du CHU Sainte-Justine 3175, Chemin de la Côte-Sainte-Catherine, Montreal, QC H3T 1C, Canada; david.morais.hs@ssss.gouv.qc.ca; 4 Laboratório de Microbiologia Geral, Universidade Federal do Vale do São Francisco, Petrolina 56304-917, Brazil; roberta.lane@univasf.edu.br; 5 Instituto Agronômico de Pernambuco, Recife 50761-000, Brazil; felix.antonio@ipa.br

**Keywords:** *Vigna unguiculata*, early response, signaling, plant defense, biotic stresses

## Abstract

*Cowpea aphid-borne mosaic virus* (CABMV) and *Cowpea severe mosaic virus* (CPSMV) threaten cowpea commercial production. This study aimed to analyze Conserved Transcriptional Signatures (CTS) in cowpea’s genotypes that are resistant to these viruses. CTS covered up- (UR) or down-regulated (DR) cowpea transcripts in response to CABMV and CPSMV mechanical inoculations. The conservation of cowpea’s UR defense response was primarily observed with the one hpi treatments, with decreased CTS representatives as time elapsed. This suggests that cowpea utilizes generic mechanisms during its early interaction with the studied viruses, and subsequently employs more specialized strategies for each viral agent. The potential action of the CTS-UR emphasizes the importance of redox balance, ethylene and jasmonic acid pathways. Additionally, the CTS-UR provides evidence for the involvement of *R* genes, PR proteins, and PRRs receptors—extensively investigated in combating bacterial and fungal pathogens—in the defense against viral inoculation. AP2-ERF, WRKY, and MYB transcription factors, as well as PIP aquaporins and MAPK cascades, also emerged as significant molecular players. The presented work represents the first study investigating conserved mechanisms in the cowpea defense response to viral inoculations, highlighting relevant processes for initial defense responses.

## 1. Introduction

Cowpea (*Vigna unguiculata*) is an important leguminous crop extensively cultivated in impoverished, hot, and arid regions across Africa, Asia, South America, and the United States of America [1]. It is valued not only for its grains with high protein content (approximately 30% protein), but also for generating earnings for low-income populations, particularly for small-scale farmers in developing countries [2].

Despite its tolerance to adverse environmental conditions, cowpea cultivation faces numerous biotic challenges. Among these, two viral pathogens, namely, *Cowpea aphid-borne mosaic virus* (CABMV) and *Cowpea severe mosaic virus* (CPSMV), pose significant threats to the crop’s productivity. CABMV has been reported to cause yield reductions of up to 85% [3], while CPSMV is associated with yield losses of up to 64% [4]. Both viruses depend on mechanical injury to plant tissues, facilitated by insect (herbivores) vectors or certain agricultural practices, to penetrate their host plants [5,6,7]. Once established, these viruses induce severe mosaic patterns on infected leaves, characterized by alternating yellow areas and patches of green [8]. Additionally, CPSMV causes leaf blade curling due to the formation of blister-like structures [8].

The plant’s defense response to bacterial and fungal infections is triggered within minutes upon the recognition of Microbial- or Pathogen-Associated Molecular Patterns (PAMPs/MAMPs) or Damage-Associated Molecular Patterns (DAMPs) by Pattern Recognition Receptor (PRR) proteins. In the context of viral infection in plants, there is a lack of studies exploring early gene expression in the host. However, two notable studies shed light on this topic. Baebler et al. [9] conducted research on susceptible (cv. Igor) and tolerant (cv. Santé) potato (*Solanum tuberosum*) cultivars infected with *Potato virus* Y. They observed that cv. Santé exhibited higher gene expression levels at 0.5 hpi (hours post-inoculation), while cv. Igor showed increased expression only after 12 hpi. This suggests that the gene expression response timing differs between susceptible and tolerant potato cultivars. 

Another study by Widyasari et al. [10] focused on soybean’s (*Glycine* max) response to *Soybean mosaic virus*. The authors found that the defense response in soybean plants began to appear in the early hours (two to eight hpi) of virus inoculation. This early response involved a complex network of transcription factors and hormonal signaling. The researchers proposed that transcriptional modulations during these early hours post-inoculation play a critical role in determining the induction of resistance or susceptibility in soybean.

Previous studies have indicated that plants tend to modulate the expression of a similar set of genes even when different viruses infect them. For example, Miozzi et al. [11] identified conserved responses of tomato (*Solanum lycopersicum*) to three distinct geminiviruses. Similarly, studying *Arabidopsis thaliana*, Rodrigo et al. [12] compared multiple expression profiles and identified several conserved responses to distinct viruses, including four members of the *Potyvirus* genus (same clade of CABMV). Notably, the mentioned study demonstrated that a certain level of conservation persists even when comparing responses to both RNA and DNA viruses [12].

Our research group recently implemented cowpea omics analyses, generating the Cowpea Genomics Consortium (CpGC). CpGC provides molecular data related to transcriptomes under abiotic or biotic stresses [13,14,15], as well as the genome sequencing of cowpea genotypes relevant in breeding programs. Among the transcriptomes available, particular attention is given to those associated with CABMV or CPSMV mechanical inoculations, which simulate the natural process of viral exposure. These bioassays, involving treatments at 1 and 16 hpi, were carefully designed to yield insights into cowpea’s response to each viral agent individually, and enable integration and comparison between them. The experimental designs employed resistant cowpea genotypes, similar treatment times, identical cultivation conditions, and viral inoculation methodologies.

Based on the mentioned scientific *status quo*, the current study aimed to explore and address several pertinent questions related to the molecular physiology of cowpea–virus interactions, such as:

- Is there a cowpea conserved transcriptional response (up- or down-regulated) to the CABMV and CPSMV mechanical inoculations at early analyzed time points (1 or 16 hpi treatments)?
- If the conservation exists, what are the critical biological processes, molecular functions, and metabolic and signaling pathways associated with the conserved response of up-regulated transcripts for a 1 or 16 hpi treatment?
- Are the up-regulated co-expressed genes widely present among Viridiplantae species?
- Based on the scrutinized data, can a tentative model of the cowpea transcriptional conserved response be constructed?

The present work represents the first study investigating conserved mechanisms in the early defense response of the cowpea to the viral inoculation processes. We present high-quality information related to the perception, signaling, and adjustment of the initial defense responses of the mentioned legume under the imposed conditions. 

## 2. Materials and Methods

### 2.1. Plant Material, Experimental Design, and Virus Mechanical Inoculation Strategy

The analyzed plant materials, experimental design (Figure S1), and virus mechanical inoculation strategy followed those set out by Ferreira-Neto et al. [14]. A brief overview of the implemented steps is presented below. 

The assays were conducted in controlled greenhouses at the Instituto Agronômico de Pernambuco (IPA; Pernambuco-Brazil) in isolated spaces for each assay, treatment, and respective controls. The IT85F-2687 [16,17] and BR-14 Mulato [18] genotypes—resistant, respectively, to CABMV (*Cowpea aphid-borne mosaic virus*) and CPSMV (*Cowpea severe mosaic virus*)—were utilized in the presented assays. The mentioned organisms were obtained from the IPA Cowpea Germplasm Active Bank and cultivated for three weeks under a natural photoperiod and temperatures between 28 and 32 °C. 

After the mentioned timeframe, trifoliolate leaves were mechanically inoculated (i.e., injured with carborundum (silicon carbide), followed by viral (CABMV or CPSMV) inoculation) [19] (Figure S1). Tissue samples were collected at 1 and 16 h post-inoculation (hpi) for each assay, along with their respective control samples (Figure S1). 

The experimental design (Figure S1) compared different cultivars and post-inoculation periods. Each combination of cultivar, post-inoculation timeframe, and respective controls had three biological replicates (Figure S1), and each replicate consisted of five plants.

The differential gene expression analysis covers the response to the virus (CABMV or CPSMV) mechanically inoculated. Plant viruses, unlike their animal-infecting counterparts, lack specific cell receptors and cannot independently initiate the infectious process as they are unable to breach the plant cell wall [20]. Consequently, in natural environments, they rely on mechanically injured plant tissues—either caused by vector organisms (herbivores) or as a consequence of specific agricultural practices—to access the target cells. Thus, we aimed to replicate the process that occurs in a natural environment. The used approach provided a robust platform to assess and elucidate the intricate gene expression changes induced by CABMV and CPSMV infection, offering valuable insights into their pathogenic mechanisms.

### 2.2. Total RNA Processing and RNA-Seq Library Synthesis

Total RNA extraction was performed using the SV Total RNA Isolation System kit (Promega, Madison, WI, USA), following the manufacturer’s protocol. The concentration, purity, and integrity of the extracted RNA were assessed using a Qubit Fluorometer (Thermo Fisher Scientific, Waltham, MA, USA), NanoDrop^®^ 2000 (Thermo Fisher Scientific, Waltham, MA, USA), and the Agilent 2100 Bioanalyzer (Agilent Technologies, Santa Clara, CA, USA), respectively. Only samples with RNA integrity number (RIN) ≥ 8.0 were selected for sequencing. 

For messenger RNA purification and cDNA library construction, the RNAm TruSeq^®^ Stranded LT-Set A kit (RS-122-2101) (Illumina, San Diego, CA, USA) was utilized, following the manufacturer’s instructions. 

Paired-end reads, with a length of 100 bp, were generated using the Illumina HiSeq 2500 system. The sequencing process involved the HiSeq^®^ Rapid PE Cluster Kit v2 (PE-402-4002), SBS Kit v2 (200 Cycle; FC-402-4021), and TruSeq^®^ Stranded mRNA LT—Set A (RS-122-2101) kits. All sequencing procedures were performed at the Center for Functional Genomics, University of São Paulo (Piracicaba, Brazil).

### 2.3. RNA-Seq Data Assembly and Differential Expression Analysis

The raw reads obtained from the sequencing were subjected to assembly using the RNA-Seq de novo Pipeline from the GenPipes project [21], developed by the McGill University and Génome Québec Innovation Center (C3G). 

The sequenced RNA-Seq libraries for CABMV and CPSMV bioassays were assembled together. Such joint assembly allowed us to obtain longer and more robust transcripts. However, differential gene expression analyses were performed independently for each assay conducted using the edgeR tool [22], integrated within the GenPipes pipeline. Transcripts exhibiting Log_2_FC values smaller than “−1” and higher than “1”, a *p*-value less than 0.05, and a false discovery rate (FDR) lower than 0.05 were considered to be differentially expressed.

### 2.4. Conserved Transcriptional Signatures (CTS) Identification

After the differential expression analysis performed for each assay, the differentially expressed transcripts were searched and four groups of “Conserved Transcriptional Signatures” (CTS) were formed. The CTS groups followed the configuration below:

- CTS-1UR (up-regulated transcripts in response to CABMV and CPSMV mechanical inoculations at one hpi treatments);
- CTS-1DR (down-regulated transcripts in response to CABMV and CPSMV mechanical inoculations at one hpi treatments);
- CTS-16UR (up-regulated transcripts in response to CABMV and CPSMV mechanical inoculations at 16 hpi treatments);
- CTS-16DR (down-regulated transcripts in response to CABMV and CPSMV mechanical inoculations at 16 hpi treatments).

### 2.5. Biological Processes and Molecular Functions Enrichment Analysis

To conduct the mentioned analysis, we employed the NeVOmics tool (Network-based Visualization for Omics; (version 1.0) [23], which enables the identification of statistically enriched biological processes and molecular functions within a specified set of genes or proteins. 

The enrichment analysis utilized a hypergeometric distribution with a significance threshold of *p* < 0.05. Additionally, false discovery rate (FDR < 0.05) correction was applied to identify GO terms that were statistically overrepresented. Each assay’s complete transcriptome data (“control vs. treatments” comparisons) were used as the background in the NeVOmics enrichment analyses. The NeVOmics tool employs a plain-text input file containing a list of proteins (identified by UniProt Entry name) obtained from the transcriptomic approach. Given the limited number of annotated proteins for cowpea in the UniProt database (https://www.uniprot.org/; accessed on 20 November 2022), we performed the annotation of the studied transcriptomes against common bean (*Phaseolus vulgaris*) proteins, a species closely phylogenetically related to cowpea [24].

To retrieve the UniProt entries for *P. vulgaris* proteins, we conducted a BLASTp search (cutoff < e^−10^) comparing the proteomes of *P. vulgaris* (from UniProt database) with the protein sequences derived from the translated cowpea RNA-Seq transcripts. We considered only the best hit for each alignment in our analysis.

### 2.6. MapMan Analysis of CTS Up-Regulated Groups

The transcriptomic analysis was integrated with metabolic and signaling pathways using MapMan [25]. Due to the absence of a mapping file specific to the cowpea reference genome in the MapMan software (version 3.5.1), we initially extracted the coding sequences of cowpea CTS up-regulated groups and subsequently utilized Mercator pipeline (http://www.plabipd.de/portal/mercator-sequence-annotation; accessed on 20 November 2022) for gene annotation and the generation of the corresponding mapping file. 

The cited sequence annotation pipeline, provided by MapMan (mapman.gabipd.org; accessed on 20 November 2022), was employed to assign MapMan Bins to the identified CTS, leveraging *P. vulgaris* proteins from Swiss-Prot and UniRef90 as references. Subsequently, the mapping file containing CTS gene expression values was imported into MapMan to analyze transcriptional regulation in metabolic pathways. 

### 2.7. CTS Mining for Jasmonic Acid and Ethylene Biosynthesis Pathways 

The results of the present work suggest the influence of jasmonic acid (JA) and ethylene (ET) biosynthesis pathways on the CTS-1UR group. Therefore—based on literature data reported for ethylene (i.e., [26,27]) and jasmonic acid (i.e., [28,29])—the biosynthesis pathways of these two phytohormones were manually reconstructed. Subsequently, the CTS-1UR members, identified by their respective EC (Enzyme Commission) numbers, were mapped onto these pathways.

### 2.8. qPCR: Setup, cDNA Synthesis, Primers Efficiency Analysis, and Relative Expression

The analyses followed the guidelines outlined in the MIQE (Minimum Information for Publication of Quantitative Real-Time PCR Experiments) [30]. A subset of target transcripts, which exhibited up-regulation in the RNA-Seq libraries, was chosen for further investigation using quantitative PCR (qPCR) to assess the transcriptomic data precisely. Detailed information regarding the reference genes employed, efficiency analysis, differential expression methodology, total RNA processing, and cDNA synthesis can be found in Ferreira-Neto et al. [14].

### 2.9. Orthology of CTS-Coding Loci in Viridiplantae Species

To explore the orthology of the cowpea CTS-coding loci with genes from other taxa, relevant information was obtained from the Phytozome (https://phytozome-next.jgi.doe.gov/; accessed on 30 November 2022) database. For this, a query was created in the PhytoMine platform (an InterMine interface to data from Phytozome), containing the cowpea gene identifiers (Vunguiculata_469_v1.1) for analysis against the *Phaseolus vulgaris* (Fabaceae) (Pvulgaris_442_v2.1), *Glycine max* (Fabaceae) (Gmax_508_Wm82.a4.v1), *Medicago truncatula* (Fabaceae) (Mtruncatula_285_Mt4.0v1), *Gossypium raimondii* (Malvaceae) (Graimondii_221_v2.1), *Salix purpurea* (Salicaceae) (Spurpurea_519_v5.1), *Populus trichocarpa* (Salicaceae) (Ptrichocarpa_533_v4.1), *Manihot esculenta* (Euphorbiaceae) (Mesculenta_305_v6.1), *Linum usitatissimum* (Linaceae) (Lusitatissimum_200_v1.0), *Prunus persica* (Rosaceae) (Ppersica_298_v2.1), *Solanum lycopersicum* (Solanaceae) (Slycopersicum_514_ITAG3.2), *Eucalyptus grandis* (Myrtaceae) (Egrandis_297_v2.0), *Capsella rubella* (Brassicaceae) (Crubella_474_v1.1), *Mimulus guttatus* (Phrymaceae) (Mguttatus_256_v2.0), *Brassica rapa* (Brassicaceae) (BrapaFPsc_277_v1.3), *Kalanchoe laxiflora* (Crassulaceae) (Klaxiflora_309_v1.1), *Theobroma cacao* (Malvaceae) (Tcacao_523_v2.1.), *Aquilegia coerulea* (Ranunculaceae) (Acoerulea_322_v3.1), *Trifolium pratense* (Crassulaceae) (Tpratense_385_v2) and *Kalanchoe fedtschenkoi* (Crassulaceae) (Kfedtschenkoi_382_v1.1) assembled and annotated genomes. The orthologous pairs were generated by the InParanoid software [31], which is implemented on the PhytoMine platform.

## 3. Results

### 3.1. Is There a Cowpea Conserved Transcriptional Response (Up-Regulated or Down-Regulated) to the CABMV and CPSMV Mechanical Inoculations at Early Analyzed Time Points (1 or 16 Hpi Treatments)? 

**Figure 1:**
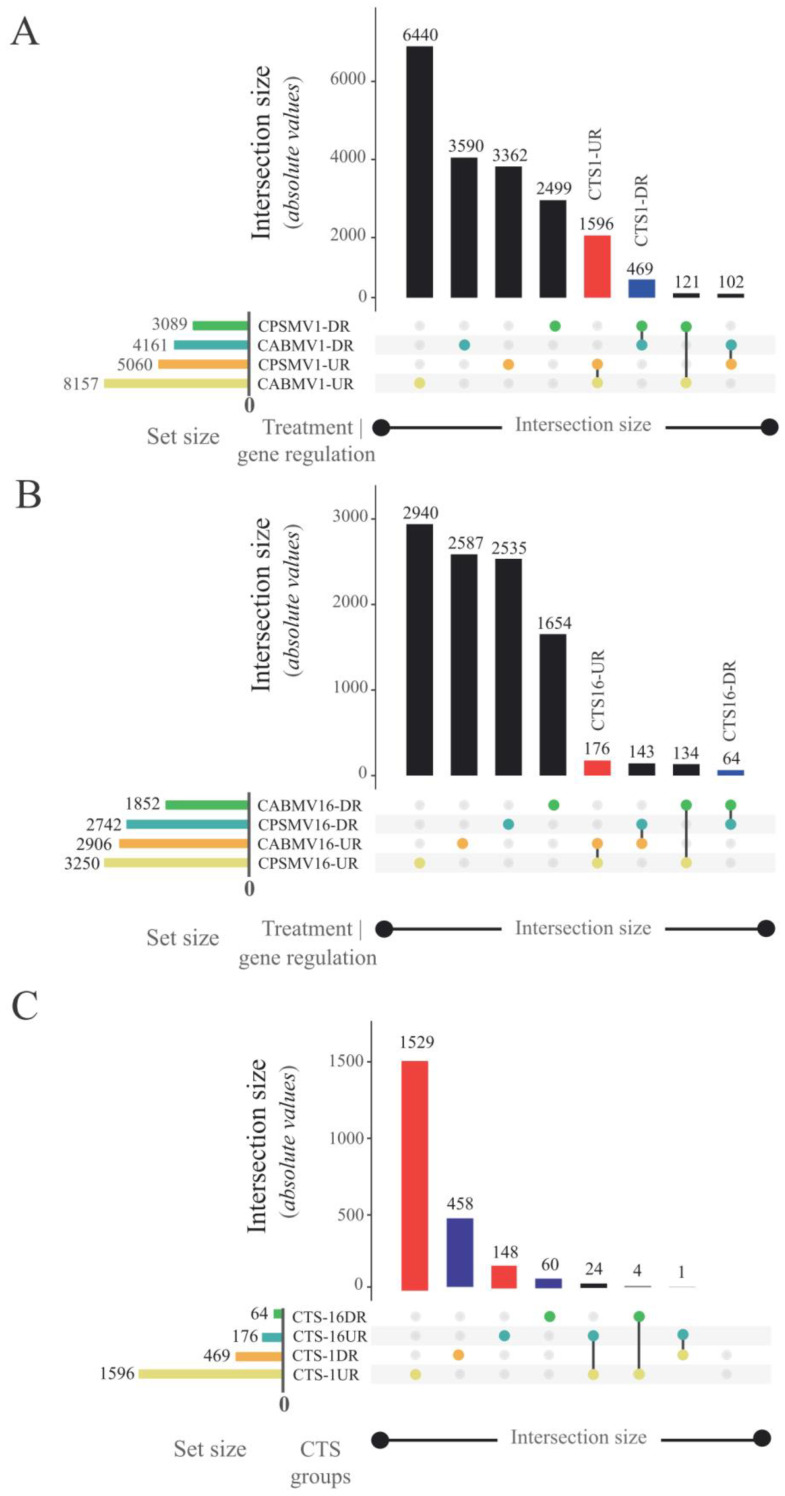
UpSet plots generated to highlight the formation of different CTS groups in response to CABMV and CPSMV mechanical inoculation. Each horizontal column represents an original dataset, and the bar charts at the top indicate the resultant dataset size after comparison with the others. Each line represents a potential intersection: filled-in cells connected by lines indicate which datasets are part of an intersection. Red and blue bars indicate up-regulated and down-regulated CTS groups, respectively. (**A**) Set of differentially expressed transcripts for the one hour post-inoculation (hpi) treatments and their resulting CTS groups. (**B**) Set of differentially expressed transcripts for the 16 hpi treatments and their resulting CTS groups. (**C**) The comparison of CTS groups of different temporal scales. Legend: CTS (Conserved Transcriptional Signatures); CPSMV1-UR group (up-regulated transcripts in the CPSMV mechanical inoculation assay at one hour post-inoculation); CPSMV16-UR group (up-regulated transcripts in the CPSMV mechanical inoculation assay at 16 hpi); CPSMV1-DR group (down-regulated transcripts in the CPSMV mechanical inoculation assay at one hpi); CPSMV16-DR group (down-regulated transcripts in the CPSMV mechanical inoculation assay at 16 hpi); CABMV1-UR group (up-regulated transcripts in the CABMV mechanical inoculation assay at one hpi); CABMV16-UR group (up-regulated transcripts in the CABMV mechanical inoculation assay at 16 hpi); CABMV1-DR group (down-regulated transcripts in the CABMV mechanical inoculation assay at one hpi); CABMV16-DR group (down-regulated transcripts in the CABMV mechanical inoculation assay at 16 hpi); CTS-1UR group (up-regulated transcripts in response to CABMV and CPSMV mechanical inoculations at one hpi treatments); CTS-1DR group (down-regulated transcripts in response to CABMV and CPSMV mechanical inoculations, at one hpi treatments); CTS-16UR group (up-regulated transcripts in response to CABMV and CPSMV mechanical inoculations, at 16 hpi treatments); CTS-16DR group (down-regulated transcripts in response to CABMV and CPSMV mechanical inoculations, at 16 hpi treatments).

The answer to this question was constructed by integrating each individual assay’s analysis. In the CPSMV mechanical inoculation assay, 101,468 unique cowpea expressed transcripts were identified. Among these, 8149 transcripts (~8.03%) showed differential expression at the one hpi treatment. Out of the differentially expressed (DE) transcripts, 5060 were up-regulated (CPSMV1-UR), and 3089 were down-regulated (CPSMV1-DR) (Figure 1A). At the 16 hpi treatment, 5992 transcripts (~5.9%) exhibited differential expression, with 3250 transcripts up-regulated (CPSMV16-UR) and 2742 transcripts down-regulated (CPSMV16-DR) (Figure 1B).

The CABMV mechanical inoculation assay identified 100,770 unique expressed transcripts. Among these, 12,318 (~12.2%) displayed differential expression at the one hpi treatment. Out of the DE transcripts, 8157 were up-regulated (CABMV1-UR), and 4161 were down-regulated (CABMV1-DR) (Figure 1A). For the 16 hpi treatment, 4758 transcripts (~4.7%) exhibited differential expression, with 2906 transcripts up-regulated (CABMV16-UR) and 1852 transcripts down-regulated (CABMV16-DR) (Figure 1B).

We specifically extracted those belonging to different CTS groups from the DE transcripts obtained in the performed assays. This action resulted in the identification of 1596 transcripts for the CTS-1UR group, 469 for CTS-1DR, 176 for CTS-16UR, and 64 for CTS-16DR (Figure 1A,B). The formation of these datasets revealed that a portion of the cowpea response to the mechanically inoculated viruses was conserved, particularly in the very early (one hpi) treatment.

Furthermore, the CTS groups corresponding to different temporal scales were compared (Figure 1C), revealing pronounced disparities. Only 24 transcripts (~1.6%) in CTS-1UR were consistently present in CTS-16UR, while no intersection was observed between the CTS-1DR and CTS-16DR groups (Figure 1C).

For a detailed list of all cowpea transcripts comprising the different CTS groups, including their modulations and functional annotations, please refer to Table S1.

### 3.2. What Are the Primary Biological Processes, Molecular Functions, and Metabolic and Signaling Pathways Associated with the Conserved Response? 

To facilitate a comprehensive understanding and improve data visualization, the question above will be systematically divided across and answered in the forthcoming sections.

#### 3.2.1. GO Enrichments Analysis for Up-Regulated CTS Groups: A Focus on Biological Processes and Molecular Functions

The distinct up-regulated CTS groups, namely, CTS-1UR and CTS-16UR, underwent analysis using the NeVOmics tool to identify enriched (i.e., statistically significant in the composition of the analyzed datasets) molecular functions (MF) and biological processes (BP).

**Figure 2:**
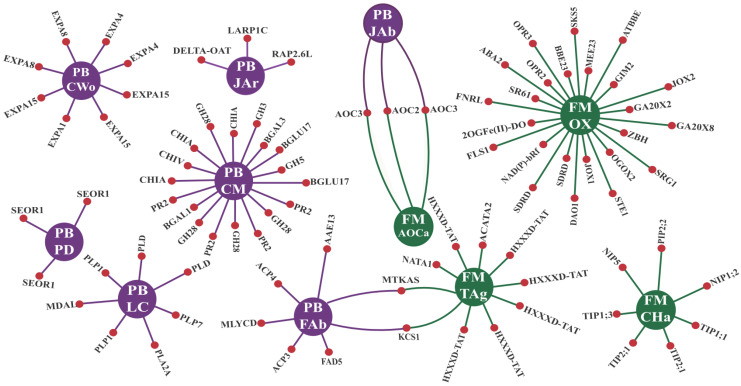
Interaction network* for CTS-1UR enriched GO terms in the MF (molecular function) and BP (biological process) categories. The central circles (in purple for BP and green for MF) represent the enriched GO term, while the side circles (in red) correspond to the proteins comprising the respective term. Legend: *Adapted from the NeVOmics tool output. CWo (plant-type cell wall organization); LC (lipid catabolic process); FAb (fatty acid biosynthetic process); PD (phloem development); CM (carbohydrate metabolic process); JAb (jasmonic acid biosynthetic process); JAr (response to jasmonic acid); AOCa (allene-oxide cyclase activity); CHa (channel activity); TAg (transferase activity, transferring acyl groups other than amino-acyl groups); OX (oxidoreductase activity); CTS-1UR group (up-regulated transcripts in response to CABMV and CPSMV mechanical inoculations, at one hpi treatments); hpi (hours post inoculation).

For CTS-1UR, four enriched GO terms for MF and seven for BP were identified, as shown in Figure 2. The enriched MF terms were associated with “transferase” and “oxidoreductase” activities, specifically “transferase activity” and “transferring acyl groups other than amino-acyl groups” (TAg), as well as “oxidoreductase activity” (OX) (Figure 2). Additionally, within the CTS-1UR group, the “allene-oxide cyclase activity” (AOCa) enriched GO term was observed (Figure 2). The transcripts encompassing this term encode key enzymes (AOC2 and AOC3) involved in jasmonic acid biosynthesis. Furthermore, the enrichment of the “channel activity” (CHa) GO term was found. The CHa term primarily consisted of transcripts encoding aquaporin proteins (AQPs) from the PIP (PIP2;2), TIP (TIP1;1, TIP1;3, and TIP2;1), and NIP (NIP1;2 and NIP5) subfamilies (Figure 2).

The enriched BP terms for CTS-1UR were associated with various plant biology aspects. Specifically, the enriched terms included “plant-type cell wall organization” (CWo) and “phloem development” (PD), highlighting processes related to plant structural components (Figure 2). Additionally, BP terms associated with the metabolism of lipids, fatty acids, and carbohydrates were enriched, such as “lipid catabolic process” (LC), “fatty acid biosynthetic process” (FAb), and “carbohydrate metabolic process” (CM) (Figure 2).

Furthermore, two enriched BP terms were associated with jasmonic acid metabolism, namely, “jasmonic acid biosynthetic process” (JAb) and “response to jasmonic acid” (JAr) (Figure 2). Within these terms, the data included protein-coding transcripts that serve as responsive elements to pathogen invasion, such as PR-2 proteins and chitinases (PR-8 and PR-11). Additionally, transcripts encoding proteins that regulate plant immune responses, as key enzymes involved in jasmonic acid biosynthesis (AOC2 and AOC3) and RAP2.6L transcription factors, were also present (Figure 2).

**Figure 3:**
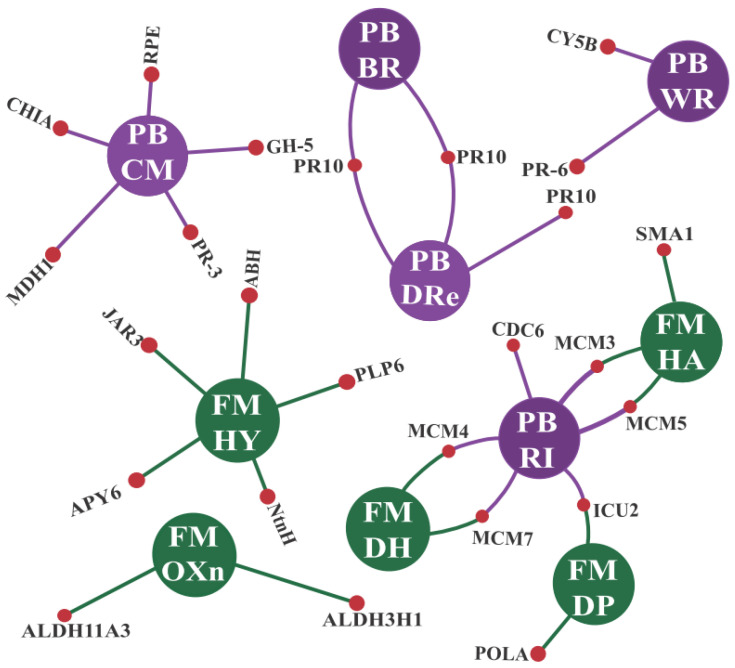
Interaction network* for CTS-16UR enriched GO terms in the MF (molecular function) and BP (biological process) categories. The central circles (in purple for BP and green for MF) represent the enriched GO term, while the side circles (in red) correspond to the proteins comprising the respective term. Legend: *Adapted from the NeVOmics tool output. RI (DNA replication initiation); WR (response to wounding); BR (response to biotic stimulus); DRe (defense response); CM (carbohydrate metabolic process); DP (DNA primase activity); DH (DNA helicase activity); HA (helicase activity); OXn (oxidoreductase activity, acting on the aldehyde or oxo group of donors, NAD or NADP as acceptor; HY: hydrolase activity); CTS-16UR group (up-regulated transcripts in response to CABMV and CPSMV mechanical inoculations, at 16 hpi treatments); hpi (hours post inoculation).

For the CTS-16UR group, enrichment analysis revealed five GO terms related to molecular functions (MF). However, these terms were biologically less informative, being associated with generic actions such as DNA replication. The terms include “DNA primase activity” (DP), “DNA helicase activity” (DH), and “helicase activity” (HA) (Figure 3). Additionally, there were terms associated with the enzymatic actions of oxidoreductases (OXn) and hydrolases (HY) (Figure 3).

Considering BP, the CTS-16UR group exhibited more informative results. Three enriched GO terms should be mentioned: “defense response” (DRe), “response to biotic stimulus” (BR), and “response to wounding” (WR) (Figure 3). Within both DRe and BR terms, transcripts encoding pathogenesis-related (PR) proteins were prominent components, with PR-10 proteins being particularly noteworthy (Figure 3). As for the enriched term WR, proteins such as PR-6 deserve special attention (Figure 3).

#### 3.2.2. CTS and Their Intricate Metabolic and Signaling Pathways 

Using the MapMan tool, we mapped all the CTS groups onto different metabolic and signaling modules related to plant biotic stress responses. For the CTS-16UR and CTS-16DR groups, detailed information can be found in Appendix S1. The number of CTS associated with the cited datasets was lower than that of its counterparts (Figure 1A,B). CTS derived from the 16 hpi treatments accounted for approximately 12% of the total CTS related to the 1 hpi treatments (Figure 1A,B). This substantial difference resulted in limited biologically informative insights from the CTS-16UR and CTS-16DR MapMan analyses. 

**Figure 4:**
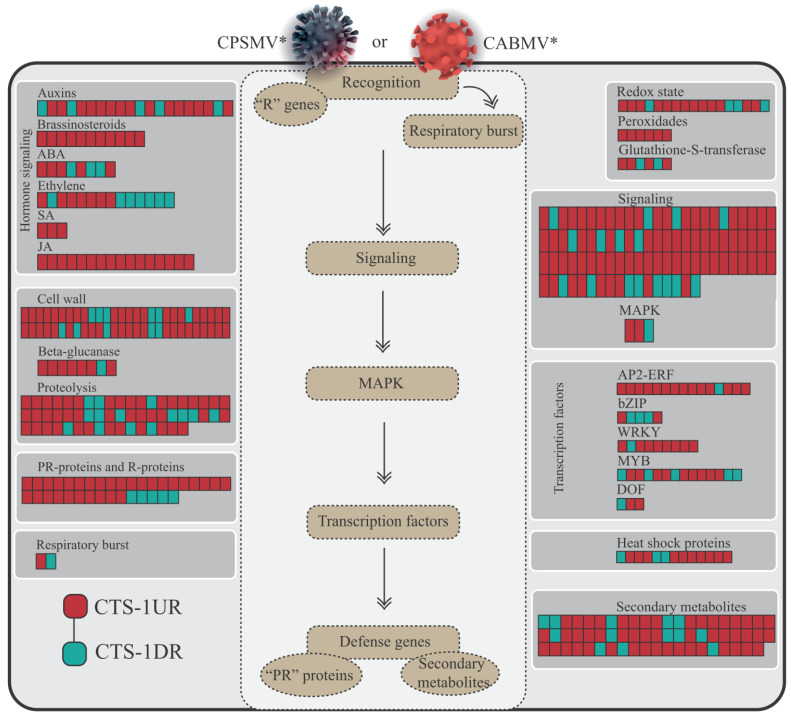
Transcripts from the CTS-1UR and CTS-1DR groups mapped onto the biotic stress response modules of the MapMan tool. Legend: * for illustrative purposes only; CTS-1UR group (up-regulated transcripts in response to CABMV and CPSMV mechanical inoculations in one hpi treatments); CTS-1DR group (down-regulated transcripts in response to CABMV and CPSMV mechanical inoculations in one hpi treatments); hpi (hours post-inoculation); colored squares/rectangles indicate different transcripts.

Regarding the CTS-1UR and CTS-1DR groups, their higher abundance provided significant insights into the similarities in cowpea’s response to CABMV and CPSMV mechanical inoculations. The resulting graphical representation (Figure 4) shows the cowpea conserved response to CABMV or CPSMV mechanical inoculations with the one hpi treatments, emphasizing the shared pathways involved.

In the following subsections, we will scrutinize important components of Figure 4, focusing on the CTS-1UR subset, highlighting the molecular actors actively participating in the conserved response under analysis.

a. Enzymes responsive to oxidative stress or involved in hormonal metabolic processes

Concerning the first category of targets, the CTS-1UR transcripts were assigned to specific MapMan modules, and among these the “Redox State” module stood out, which contained annotations for TRX2, TRX1, PDI2, APX3, and others; so too did the “Peroxidases” module, which included PRX52, PRX25, PRX64, among others, and the “Glutathione-S-Transferases” module, which included GSTU25, GSTU8, GSTU20 enzymes (Figure 4, Table S2). Additional analyses conducted in the CTS-1UR group, as outlined in Table S2, revealed, along with the mentioned ROS scavenging enzymes, the presence of Rboh-encoding transcripts (an enzyme involved in a reactive oxygen species (ROS) biogenesis).

Regarding the second category, the conserved responses were characterized by the transcriptional up-regulation of enzymes involved in jasmonic acid (JA) biosynthesis, such as LOX1, LOX2, AOC3, AOC4, OPR1, OPR2, and OPR3. Similarly, ethylene (ET) biosynthesis enzymes—including ACS1, ACS6, and ACO3—were also identified. Furthermore, proteins responsive to various other phytohormones were constituents of the CTS-1UR group, including abscisic acid (ABA) (e.g., ABA2, HVA22F), salicylic acid (SA) (e.g., BSMT), brassinosteroids (BR) (e.g., NILR1, BIM1, DWARF 4, BR6OX2), and auxin (e.g., ILL6, SAUR, ARF, GH3, IAR3) (Figure 4, Table S2).

b. Signaling proteins and transcription factors

In total, 76 CTS-1UR (indicated by red squares in Figure 4) were identified and mapped to the signaling module. Among the identified elements, transcripts encoding Receptor-like kinases (RLKs), MAPKK kinases (MAPKKKs), Ca^2+^-dependent protein kinases (CPKs or CDPKs), calmodulin-like proteins (CMLs), and calmodulin-binding proteins (CaM-binding) were prominent (Figure 4 and Table S2).

Within the RLK elements, the most abundant in the signaling module, it is noteworthy to mention the CTS-1UR transcripts encoding PRRs, which are commonly involved in PAMPs, i.e., perception of bacteria or fungi (Figure 4 and Table S2). These PRRs included PXY/TDR-CORRELATED (PXC1), SRF8, LYSM RLK1-INTERACTING KINASE 1, BAK1-interacting receptor-like kinase 1 (BIR1), ER-LIKE1 (ERL1), Cys-rich RLK (CRK10, CRK42, CRK10, CRK29, CRK2), Lectin-RLK (LecRK-IV.1, LecRK-S.2, LecRK-S.4, LecRK-S.7), and BRASSINOSTEROID-SIGNALING KINASE 3 (BSK3).

Regarding transcription factors (TFs), members of the AP2-ERF, WRKY, and MYB families were predominant in the initial defense response, accounting for 67% of the TFs within the CTS-1UR group (Figure 4 and Table S2).

c. *R* genes and PR proteins

In the case of genes encoding R proteins belonging to the NBS-LRR family, four CTS-1UR transcripts were identified, consisting of two TIR-NBS-LRR and two CC-NBS-LRR members (Figure 4 and Table S3). This observation suggests the very early involvement of these molecular players in the conserved cowpea response to the CABMV or CPSMV mechanical inoculations.

As for PR proteins, a total of 26 CTS-1UR transcripts were discovered. These proteins belonged to various PR families, including PR-2 (β-1,3-glucanases), PR-3 (chitinases), PR-5 (thaumatin-like proteins), PR-9 (peroxidases), and PR-10 (Bet v1-like proteins) (Figure 4 and Table S3).

#### 3.2.3. CTS and Phytohormones: An Emphasis on the JA and ET Biosynthesis Pathways

Previous analyses demonstrated that three gene ontology (GO) terms associated with jasmonic acid (JA) were enriched in the CTS-1UR group (Figure 2). Additionally, the MapMan analysis of the dataset used, specifically focusing on the hormonal signaling module, further emphasized the processes related to JA and ethylene (ET) biosynthesis (Figure 4 and Table S2).

**Figure 5:**
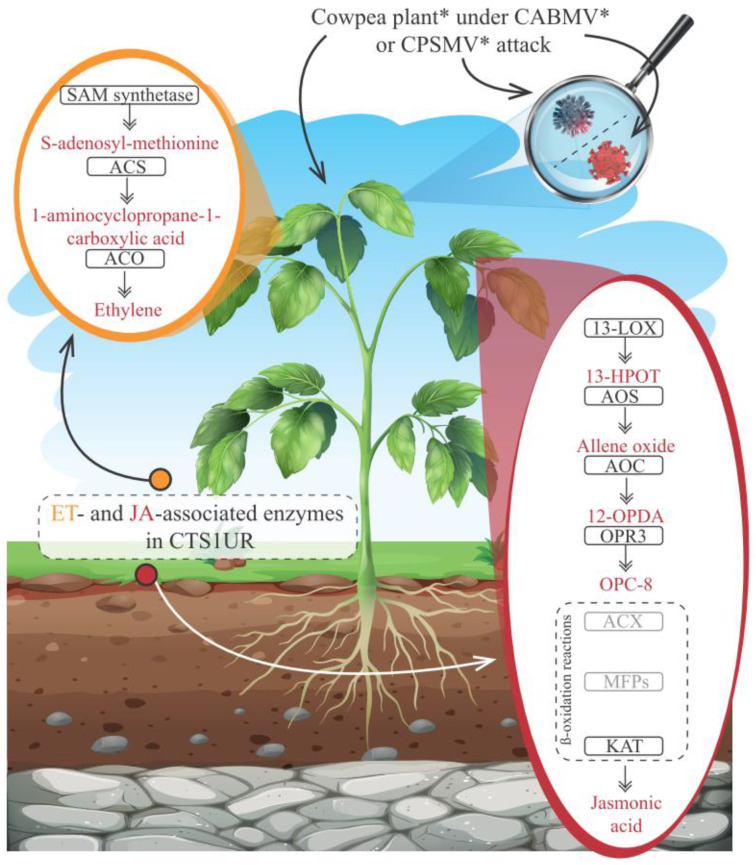
Schematic representation of a cowpea plant* under attack by CABMV* or CPSMV*, highlighting the ethylene (ET) and jasmonic acid (JA) biosynthesis pathways and emphasizing the enzymes (inside black rectangles) belonging to the CTS-1UR group. Legend: *for illustrative purposes only; enzymes inside gray rectangles showed constitutive expression in the both analyzed assays; ET (Ethylene); JA (jasmonic acid); SAM synthetase (S-adenosylmethionine synthetase); ACS (1-aminocyclopropane-1-carboxylate synthase); ACO (1-aminocyclopropane-1-carboxylate oxidase); 13-LOX (13-lipoxygenase); 13-HPO (13(S)-linolenic acid hydroperoxide); AOS (allene oxide synthase) AOC (allene oxide cyclase); ACX (acyl-CoA-oxidase); 12-OPDA (cis (+)-12-oxo-fitodienóico)); OPR3 (OPDA reductase); OPC8 (3-oxo-2-(2-pentenyl)-cyclopentane-1-octanoic acid); MFP (multifunctional proteins); KAT (L-3-ketoacyl-CoA-thiolase); CTS-1UR group (up-regulated transcripts in response to CABMV and CPSMV mechanical inoculations, at one hpi treatments); hpi (hours post inoculation).

Considering the observed potential influence of JA in this study, and the well-known synergistic role between JA and ET, we manually reconstructed their biosynthesis pathways based on data form the literature. Subsequently, the transcripts belonging to the CTS-1UR group were analyzed in the mentioned context (Figure 5; Table S4).

The ET biosynthesis pathway relies on the activities of three key enzymes: S-adenosylmethionine synthetase (SAM synthetase), 1-aminocyclopropane-1-carboxylate synthase (ACS), and 1-aminocyclopropane-1-carboxylate oxidase (ACO) [26,27] (Figure 5). All these enzymes were identified in the CTS-1UR group (Figure 5 and Table S4). This suggests that the ET biosynthesis pathway represents a conserved transcriptional signature in cowpea’s response to CABMV or CPSMV mechanical inoculations. 

On the other hand, JA biosynthesis involves the activity of seven enzymes (Figure 5), as described by Wasternack and Song [29]. Within the CTS-1UR group, transcripts encoding five of these key players were identified, namely, 13-lipoxygenase (13-LOX; 1), allene oxide synthase (AOS; 2), allene oxide cyclase (AOC; 4), OPDA reductase 3 (OPR3; 11), and L-3-ketoacyl-CoA-thiolase (KAT; 2) (see Figure 5 and Table S4). The two missing enzymes (gray rectangles in Figure 5) exhibited constitutive expressions in both examined assays.

Based on the presented evidence, transcriptional indications (Figure 5) suggest that the ET and JA biosynthesis pathways exhibited an up-regulated conserved transcriptional response in the different studied accessions upon CABMV or CPSMV mechanical inoculations. This conservation was particularly evident in the CTS-1UR group, transcriptionally suggesting that these hormones played significant roles in the early defense response to viral exposure.

#### 3.2.4. qPCR Validation of the CTS Expression

**Figure 6:**
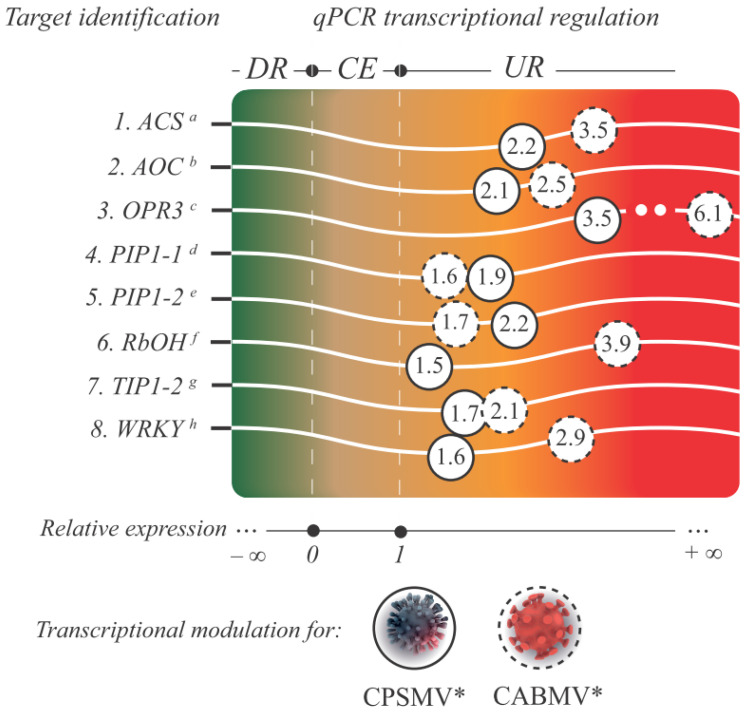
A gradient chart is presented, showcasing the analyzed targets from the CTS-1UR group, showing their respective functional annotations and IDs (superscript letters). The color gradient represents the relative expression value, indicated by the numbers inside the circles, for each target and viral mechanical inoculation assay. Legend: * for illustrative purposes only; circles with dashed lines represent the relative expression levels of the target transcripts from the one hpi treatment in the CABMV mechanical inoculation assay, while circles with solid lines represent the same for the CPSMV mechanical inoculation assay. Targets displaying a relative expression value below zero and *p* < 0.05 are classified as down-regulated (DR), those between zero and one with *p* ≥ 0.05 are considered constitutively expressed (CE), and those greater than one with *p* < 0.05 are identified as up-regulated (UR); ^a^ Vung164007|c0_g1_i1 (ACS: 1-aminocyclopropane-1-carboxylate synthase); ^b^ Vung81738|c0_g1_i1 (AOC: allene oxide cyclase); ^c^ Vung115541|c1_g5_i8 (OPR3: OPDA reductase); ^d^ Vung136249_c0_g4_i2 (PIP1-1: aquaporin PIP1-1); ^e^ Vung47464_c4_g4_i1 (PIP1-2: aquaporin PIP1-2); ^f^ Vung150570|c0_g1_i7 (Rboh: Respiratory burst oxidase homolog); ^g^ Vung59787_c1_g1_i4 (TIP1-2: aquaporin TIP1-2); ^h^ Vung50795|c1_g1_i3 (WRKY: WRKY transcription factor); CTS-1UR group (up-regulated transcripts in response to CABMV and CPSMV mechanical inoculations, with one hpi treatments); hpi (hours post-inoculation).

To evaluate the reliability and robustness of the RNA-Seq gene expression data, a subset of target transcripts from the CTS-1UR group (Figure 6) underwent additional qPCR analysis. In this step, the expression data of eight transcripts (Figure 6) associated with different protein classes were validated: ACS, AOC, OPR3, PIP1-1, PIP2-1, RbOH, TIP1-2, and WRKY.

The primer pairs designed for the target transcripts and reference genes exhibited amplification efficiencies ranging from 90.64 to 107.80% (Table S5). The specificity of the qPCR assays was confirmed by the presence of a single peak in the melting curves. Importantly, all eight qPCR-validated targets displayed statistically significant up-regulation under one hpi treatments, corroborating the findings obtained from the RNA-Seq analysis (Figure 6). These results highlight the generated RNA-Seq libraries’ high quality, and underscore the robustness of the statistical analyses employed in this study. The alleged effects of some validated CTS on cowpea defense are presented in various subitems within the “Discussion” section.

### 3.3. Are the Cowpea CTS-Encoding Genes Widely Distributed among Viridiplantae Species?

**Figure 7:**
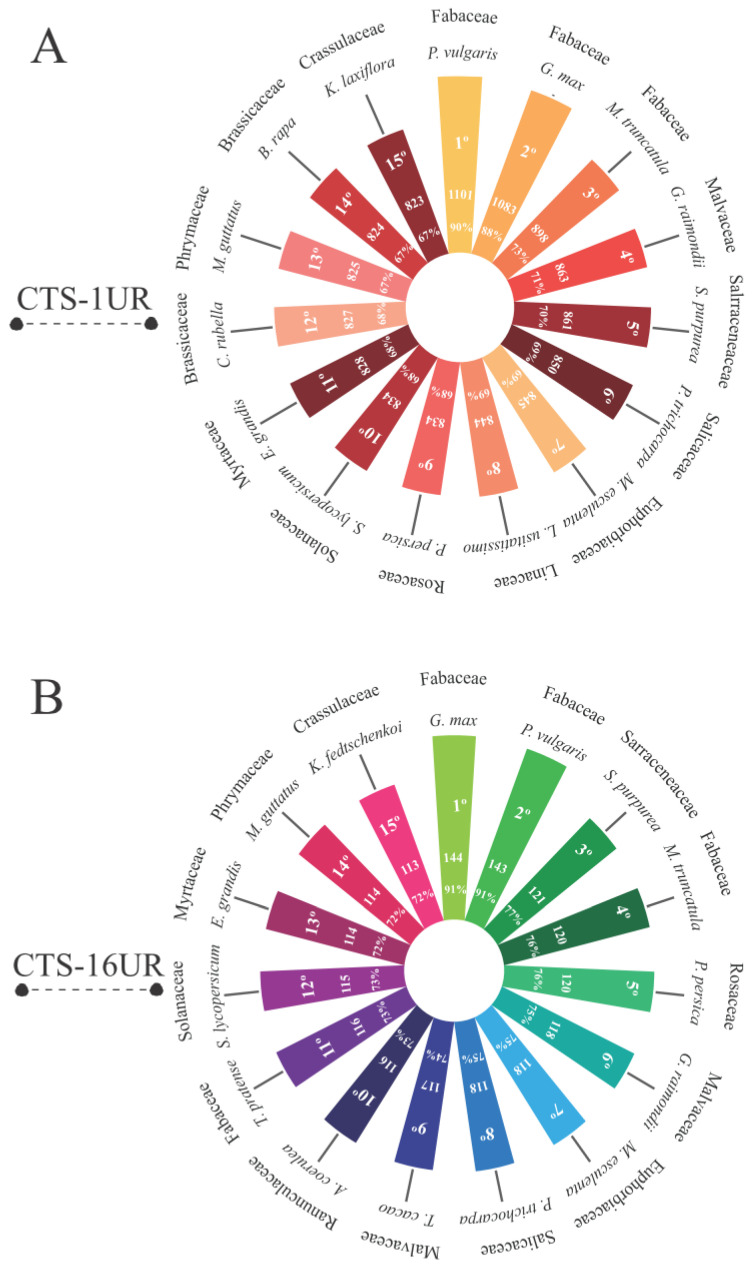
The orthology relationships of cowpea CTS-anchoring loci for CTS-1UR (**A**) and CTS-16UR (**B**) are presented, indicating the analyzed species and their respective families, along with the absolute quantity and percentage of the conserved loci among the studied organisms. Legend: CTS-1UR (group of up-regulated transcripts in response to CABMV and CPSMV mechanical inoculations, under 1 hpi treatments); CTS-16UR (group of up-regulated transcripts in response to CABMV and CPSMV mechanical inoculations, under 16 hpi treatments); hpi (hours post-inoculation).

An orthology analysis was conducted on the loci encoding CTS-1UR and CTS-16UR transcripts in cowpea, considering various species within the Viridiplantae group. The study revealed the highest abundance of orthologous pairs between cowpea and other Fabaceae species, including *P. vulgaris*, *Glycine max*, and *Medicago truncatula* (Figure 7A,B). In total, 90% of the CTS-encoding genes in cowpea had orthologs with common bean (Figure 7A,B). Moreover, a significant number of orthologs (ranging from 67% to 79%) were also identified in distantly related species belonging to families such as Salicaceae, Malvaceae, Brassicaceae, Rosaceae, Euphorbiaceae, Linaceae, Solanaceae, Myrtaceae, Phrymaceae, and Crassulaceae (Figure 7A,B).

### 3.4. Based on the Scrutinized Data, Can a Tentative Model of the Cowpea Transcriptional Conserved Response Be Constructed? 

The data derived from the CTS-1UR group allow us to propose a preliminary model describing cowpea’s transcriptional conserved response for the CABMV and CPSMV mechanical inoculations. The function of each highlighted molecular player in the present item will be thoroughly examined and referenced in the Discussion section. 

**Figure 8:**
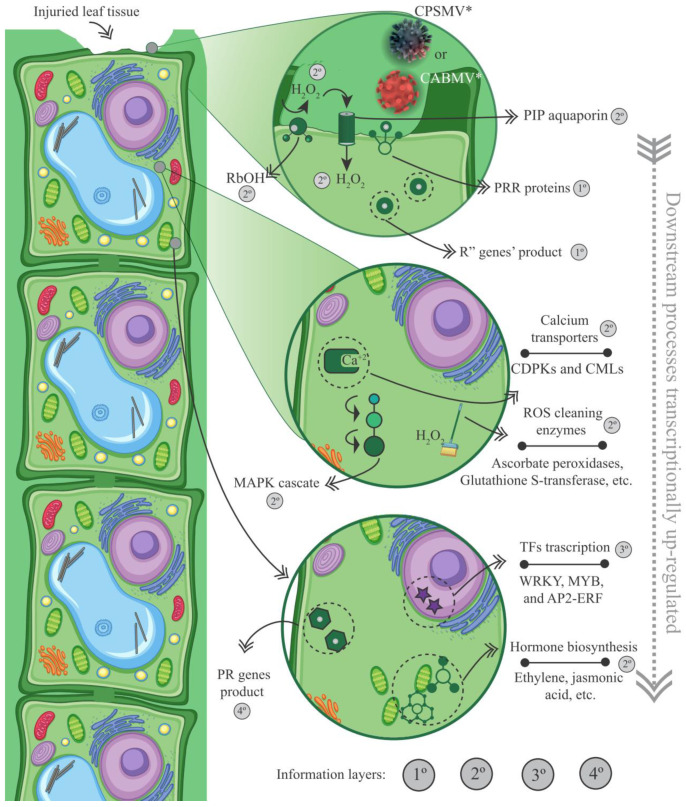
Model representation of the plant cellular environment displaying the first, second, third, and fourth information layers obtained from up-regulated conserved transcriptional signatures with the one hpi treatment (CTS-1UR group). Legend: * for illustrative purposes only; PRRs (pattern recognition receptors); CDPK (Ca^2+^ dependent protein kinases); CML (calmodulin-like proteins); Rboh (respiratory burst oxidase homolog); PIP (plasma membrane intrinsic protein); ROS (reactive oxygen species); TFs (transcription factors); PRs (pathogenesis-related proteins); CTS-1UR group (up-regulated transcripts in response to CABMV and CPSMV mechanical inoculations, with one hpi treatments); hpi (hours post-inoculation).

With the one hpi treatments, the cowpea genotypes demonstrated active engagement in the up-regulation of PRRs (membrane receptors) and *R* genes (intracellular receptors) (Figure 4 and Figure 8, Tables S2 and S3). Together, these two protein classes comprise the plant’s innate immune system, which is responsible for pathogen perception, thereby functioning during the very initial stages of the plant’s counterattack. Considering the CTS-1UR dataset, the cited molecular actors serve as the first layer of information in our preliminary model (Figure 8).

In the CTS-1UR analysis, aside from components related to the innate immune system, we also identified elements associated with signaling processes. These molecular actors constitute the second layer of information in our transcriptional tentative model (Figure 8). Among the signaling pathway components, it is suggested that ROS, particularly H_2_O_2_, may play significant roles during the very early stage under investigation. The enzyme Rboh, involved in H_2_O_2_ production, was found in the CTS-1UR dataset (Figure 6 and Figure 8, and Table S2). H_2_O_2_ has been proposed to enact multiple functions in plant defense against pathogens. Additionally, PIP membrane proteins of the aquaporin family were also identified in the CTS-1UR dataset (Figure 6 and Figure 8). These proteins may be associated with transmitting the apocytoplasmic H_2_O_2_ signal (Figure 8).

Despite being important signaling players, it is crucial to maintain a balance between ROS production and scavenging in the plant cell. This equilibrium can be maintained by enzymes such as ascorbate peroxidases, thioredoxins, peroxiredoxins, and glutathione-S-transferases, which were significant constituents of the CTS-1UR dataset (Figure 4 and Figure 8; Table S2).

Continuing within the realm of signaling actions, several CTS-1UR transcripts encoding Ca^2+^ sensors (Figure 4 and Figure 8; Table S2), including CDPKs and CMLs, were found, suggesting the involvement of Ca^2+^ as a secondary messenger. Furthermore, MAPKs also emerged as relevant components in the CTS-1UR dataset (Figure 4 and Figure 8).

To conclude the potential molecular signaling actions, several enzymes involved in JA or ET biosynthesis were identified in the CTS-1UR group (Figure 2, Figure 5, Figure 6 and Figure 8). The mentioned phytohormones are known to regulate numerous signaling pathways associated with defense mechanisms. Jasmonates are pivotal in coordinating responses against insect and arthropod herbivores that cause mechanical injury to plant tissues. ET can synergistically interact with JA in modulating stress and developmental responses. The disruption of ET responses in plants has been reported as an attack mechanism for potyviruses [32].

In our tentative model, regarding the third layer of information (Figure 8), it is important to emphasize the significance of transcription factors responsible for activating target genes. In the CTS-1UR group, several transcription factors belonging to different families, such as MYB, WRKY, and AP2-ERFs, were identified (Figure 4, Figure 6 and Figure 8). Among these, MYB, WRKY, and AP2-ERFs were the most abundant.

Moving on to the fourth and last layer of information, we have PR genes (Figure 4 and Figure 8, and Table S3). These genes encode proteins initially discovered in tobacco plants infected by the *Tobacco mosaic virus* (TMV). After viral mechanical inoculations, it has been reported that PR proteins accumulate in both infected and non-infected organs, effectively preventing further virus propagation.

## 4. Discussion

### 4.1. General Aspects of the Cowpea Conserved Response to CABMV and CPSMV Mechanical Inoculations

The comparative analysis carried out in this study allowed for the identification of identical transcripts with similar transcriptional regulation in both bioassays. The CTS investigation provided a comprehensive understanding of the conserved defense mechanisms employed by cowpea in response to CABMV and CPSMV mechanical inoculations. 

The response conservation was predominantly observed in very early time points (1 hpi treatments, particularly in CTS-1UR group), and it diminished over the utilized timeframe (16 hpi treatments) (Figure 1). Analogs were reported for resistant and susceptible potato (*S. tuberosum*) varieties inoculated with two different strains of *Potato virus* Y (Potyviridae family) [33]. It was found that a substantial number of similar genes exhibited differential expression at the very early time point (4 hpi), while a more specific response was observed at a later one (10 hpi) [33]. These findings, along with our results, emphasize that as the duration of treatment increases, the plant’s response becomes more specific to each mechanically inoculated virus and/or dependent on the studied genotypes.

In another comparative context, it has been observed that the isoforms’ composition within the CTS groups varied significantly between the treatment at 1 hpi (e.g., CTS-1UR group) and the treatment at 16 hpi (e.g., CTS-16UR group). Only a small percentage, approximately 1.6%, of the mentioned sets exhibited any similarity. This indicates that the conserved response specificity displayed across the implemented treatments is both quantitative (CTS-16UR constitutes roughly 10% of the total CTS elements found in CTS-1UR) and qualitative (the isoforms present in CTS-1UR and CTS-16UR are predominantly distinct from each other). Consequently, it becomes imperative to investigate the constituents of CTS (particularly the up-regulated groups) in each treatment to comprehend the strategies employed by cowpea in combating the studied viruses.

### 4.2. Enriched Biological Processes and Molecular Functions Unveiled Some Cowpea Molecular Weapons to Combat CABMV and CPSMV

Within the enriched molecular functions of the CTS-1UR group, a highly informative dataset, aquaporin-encoding genes, including TIPs, PIPs, and NIPs, has emerged as significant. These aquaporins are commonly associated with water and solute transport [34]. The impact of this particular protein group on the plant’s response to viral agents has been documented in the scientific literature. For instance, in tomato plants (*S. lycopersicum*) resistant to *Tomato yellow leaf curl virus*, higher levels of TIP1;1 transcripts were observed compared to susceptible ones [35]. Furthermore, TIPs silencing compromised the defense mechanisms of the studied plants, making resistant accessions susceptible [35]. Regarding PIPs, it has been observed in Arabidopsis that they facilitate the transport of apoplastic H_2_O_2_ into the cytoplasm. H_2_O_2_ synthesis is induced by PAMPs [36]. It is a relatively stable ROS, and its production in response to pathogenic attacks is carried out by the enzyme RboH [37], which is also a component of CTS-1UR. There have been no previous reports on NIP aquaporins’ involvement in response to viral mechanical inoculation. 

Another prominent group of proteins was PR proteins, which are components of the enriched terms “carbohydrate metabolic process” (in CTS-1UR) and “defense response”, “response to biotic stimulus”, and “response to wounding” (in CTS-16UR). Some PR families identified in our data, such as PR-2 and PR-10 [38], have documented action against *Tobacco mosaic virus*. The antiviral activity of PR-10 has also been demonstrated in sugarcane, as observed by Peng et al. [39]. These authors found that PR-10 proteins were up-regulated in resistant plants in response to *Sorghum mosaic virus*, while being repressed in susceptible ones. Additionally, other studies have shown the involvement of plant PR proteins, such as PR-2 [40], in the response to mechanical injury.

### 4.3. JA and ET Emerged as Prominent Players in the Cowpea Conserved Response to CPSMV and CABMV Mechanical Inoculations 

Our findings reveal the transcriptional up-regulation of JA biosynthesis in the one hpi treatment. It is presumed that alterations in the JA endogenous levels may play a significant role in the cowpea conserved response to CPSMV and CABMV mechanical inoculations.

JA and its derivatives, collectively known as jasmonates (JAs), are recognized for their dual role in signaling biotic and abiotic stress responses [41]. The activation of JA biosynthetic enzymes, such as LOXs and AOS, leads to a local and systemic increase in JAs levels in response to mechanical injury [42,43,44]. In the biological agent’s context, *Vigna mungo* plants overexpressing genes encoding AOS and AOC enzymes showed resistance against *Mungbean yellow mosaic India virus* [45]. JAs activation triggered systemic resistance by stimulating various families of transcription factors and defense-related genes [45].

The ET phytohormone, in turn, which presented the three key enzymes involved in its biosynthesis within the CTS-1UR dataset (Figure 5), not only mediates responses to mechanical injuries [46], but also plays a role in signaling networks associated with plant resistance against viruses. The process begins with the induction of ET biosynthesis [47]. ACO and ACS enzymes are involved in the antiviral response in various plants, including potato, pepper (*Capsicum annuum*), common bean (*P. vulgaris*), and *A. thaliana* [48,49,50]. Additionally, in transgenic *A. thaliana* plants, the overexpression of SbSAMS (a *Solanum brevidens* SAM synthetase) led to the expression of genes responsive to mechanical injuries [51].

### 4.4. The Crucial Role of Redox Balance, Other Phytohormones, Signaling Pathways, R Genes, and Some Transcription Factors Groups

As mentioned earlier, the CTS-1UR dataset included Rboh-encoding transcripts, which are involved in producing H_2_O_2_, a relevant ROS. Despite their significance as intracellular signaling molecules, ROS, when present in excessive concentrations, can be detrimental and lead to cellular damage [52].

In the mentioned dataset, transcripts associated with GO terms such as “Redox State”, “Peroxidases”, and “Glutathione-S-transferases” were also significant components. These terms suggest the importance of ROS scavenging-related processes under the analyzed conditions. Our transcriptomic data provide evidence that maintaining a balance between ROS production and scavenging is a crucial and conserved response of cowpea to CPSMV and CABMV mechanical inoculations. The mentioned balance may facilitate efficient defense through effective signaling mechanisms.

In addition to those already mentioned, other phytohormones also play a significant role in plant immunity, as revealed by the CTS-1UR MapMan analysis. Transcripts associated with the biosynthesis or signaling pathways of salicylic acid (SA), auxin, abscisic acid (ABA), and brassinosteroids (BRs) were found in the cited dataset. SA is recognized as a critical regulator of resistance responses against viral pathogens [53]. It plays a crucial role in hypersensitive response (HR) and the establishment of systemic acquired resistance (SAR) [54,55,56]. In its intricate role, ABA can exert a dual influence on plant defense against fungal and bacterial pathogens. However, when it comes to viral infections, its impact seems to be focused solely on augmenting the plant’s antiviral defense, as evidenced by multiple studies (reviewed by [57]). Remarkably, two distinct ABA-dependent defense mechanisms have been unveiled in plants to combat viral invasions, namely, the callose deposition at plasmodesmata and the intricate RNA silencing pathway [57]. In contrast, auxins seem to have no direct implications for antiviral responses in plants [57]. The presence of transcripts related to this phytohormone in the CTS-1UR information group may be associated with the response to mechanical damage or represent a specificity found in the analyzed species. 

Within the CTS-1UR dataset, diverse transcripts encoding RLKs were also identified. These proteins function as PRRs located in the plasma membrane and are responsible for recognizing PAMPs. The successful recognition of pathogenic signatures by PRRs initiates a cascade of downstream signaling events that trigger plant defense responses, leading to the establishment of PAMP-triggered immunity (PTI) [58]. Several studies have indicated the involvement of plant RLKs in the viral components’ recognition. In these interactions, viral components act as PAMPs, inducing typical PTI responses [59]. Kundu et al. [60] have suggested that RLKs function as an “early warning system” triggered by pathogen recognition, serving as an initial barrier that enhances the host’s ability to prevent pathogen establishment. 

Additionally, transcripts coding for molecular players involved in various signaling pathways, such as calcium-mediated signaling and MAPK signaling cascades, were up-regulated in the one hpi treatment. The scientific literature has reported that the plant host utilizes calcium signaling as a mechanism to effectively combat viral invasion. Viral infections can induce various stress-related injuries, leading to an excessive calcium influx in some plant cell components. This calcium influx can result in different forms of cell death, such as necrosis, apoptosis, and autophagic cell death. These cellular demise processes serve as crucial barriers that hinder the spread of the virus to adjacent cells and surrounding tissues (for a comprehensive review, see [61]).

The MAPK signaling pathway, in turn, plays a positive role in antiviral plant resistance by regulating the expression levels of PR [62] and *R* genes [63]. Studies have demonstrated that the overexpression of specific MAPKs can enhance plant resistance against viral pathogens. For example, the overexpression of MPK17 in transgenic tobacco (*Nicotiana tabacum*) plants led to increased resistance against viruses such as *Cucumber mosaic virus* and *Potato virus* Y [64].

*R* gene-mediated resistance stands as one of the more extensively investigated mechanisms to combat bacterial and fungal pathogens, often accompanied by the hypersensitive response (HR), and has also demonstrated its efficacy in combating viral infections. Upon encountering plant–virus interactions within a single cell, activating an *R* gene can instigate the HR, swiftly eliminating infected cells and restricting viral invasion [65].

Lastly, the presence of AP2-ERFs, WRKYs, and MYBs, which are important TFs, was notable in the CTS-1UR dataset. TFs are critical players in the entire response process analyzed here, as they directly activate the transcription of target genes. Transcriptional regulation by TFs is crucial for establishing plant defense and associated responses during viral infections. AP2-ERFs, WRKYs, and MYBs are known for their prominent roles in combating plant viruses. The detailed mechanisms through which they act against viral pathogens were extensively reviewed by Viswanath et al. [66].

### 4.5. Conservation Encompasses Not Only the Target Genes Transcription but Also the Preservation of CTS-Anchoring Gene Loci in Viridiplantae

Orthology analysis between cowpea CTS-coding genes and genomes from various species across different Viridiplantae families revealed a substantial orthology index among the studied organisms, highlighting the significance of these genes within the studied clades. The highest number of orthologs was observed within the Fabaceae family, to which cowpea belongs. Among leguminous plants, a higher number of orthologs was found between cowpea and common bean. These species share a close relationship, exhibiting a high degree of synteny [67,68]. Further investigations into the impact of these orthologous genes may uncover novel insights and provide new contributions to plant biology comprehension under stresses.

## 5. Conclusions 

The CTS identification and analysis have provided valuable insights into the conservation of cowpea defense responses against CABMV and CPSMV mechanical inoculations. The multifunctional role of CTS transcripts (critical in cowpea’s defense against the two studied pathogens) and their genomic conservation across different analyzed species make them significant biotechnological targets, particularly for legumes.

The conservation of cowpea’s defense response was predominantly observed with the one hpi treatment, with a decrease in the number of isoforms of formed CTS groups as time progressed. This suggests that cowpea initially employs generic mechanisms in the early interaction with different viruses, but adopts more specialized measures for each viral agent or leverages genetic individuality as the response progresses. 

This study highlighted CTS of molecular actors already known in the response to different pathogens, although not previously analyzed using the employed experimental design. The results based on the searched CTS emphasize the importance of redox balance, indicating the significance of ROS production and scavenging. Additionally, our work provides potential evidence for the involvement of *R* genes, PR proteins, and PRRs receptors, which have been extensively investigated in combating bacterial and fungal pathogens in the defense against viruses. Transcription factors from the AP2-ERF, WRKY, and MYB families, as well as the PIP aquaporins and MAPK cascades, also emerged as significant components of CTS-1UR.

Finally, our results highlight the importance of transcripts encoding enzymes from phytohormones biosynthetic pathways, particularly ET and JA, in the most informative dataset (CTS-1UR). The CTS-1UR group allowed us to propose a tentative transcriptional model of cowpea’s conserved response to the studied conditions. This model encompasses key molecular players that can be explored for their functional characterization and potential applications in genetic improvement through biotechnological interventions. A further investigation is warranted to delve into their specific roles and implications.

## Data Availability

The datasets generated for this study can be found in the NCBI BioProject (https://www.ncbi.nlm.nih.gov/bioproject/), with the following accession numbers: CABMV mechanical inoculation|BioSample ID: SAMN15763372; CPSMV mechanical inoculation|BioSample ID: SAMN15774013.

## Supplementary Materials

The following supporting information can be downloaded at: https://www.mdpi.com/article/10.3390/life13081747/s1. Table S1: Functional annotation of transcripts comprising the four CTS (CTS-1UR, CTS-1DR, CTS-16UR, CTS-16DR) groups obtained from cowpea response to CABMV and CPSMV mechanical inoculations. Table S2: CTS (CTS-1UR and CTS-1DR) mapped onto the biotic stress response modules of the MapMan tool. Table S3: CTS encoding *R* genes and pathogenesis-related proteins. Table S4: CTS mapped onto the biosynthesis and signaling pathways of the phytohormones ethylene and jasmonic acid. Table S5: Reference genes and target CTS used in the relative expression analysis, presenting the primer pair sequences, annotation, respective CTS group, and primer pair efficiency values. Appendix S1: Graphical representation of transcripts belonging to the CTS-16UR and CTS-16DR groups, mapped onto the biotic stress response modules of the MapMan tool. Figure S1: Graphical representation of the experimental design employed in the present study for each genotype of cowpea used. Legend: hpi (hours post-inoculation); BR (biological replicate).
