# Supplementary material for: Unlocking Cowpea’s Defense Responses: Conserved Transcriptional Signatures in the Battle against CABMV and CPSMV Viruses
Source: Life (Basel). 2023 Aug 15;13(8):1747. doi: 10.3390/life13081747 (PMC10455494; doi:10.3390/life13081747)
Supplement: Supplementary file 1 [file life-13-01747-s001.zip › ok_Appendix_S1.pdf]

Regarding the CTS-16UR and CTS-16DR datasets, MapMan tool analysis yielded limited information (Figure S1) in comparison to the CTS-1UR and CTS-1DR datasets. Specifically, for the CTS-16UR group, only a few members of the analyzed modules exhibited conserved up-regulation. This fragmented information prevented us from obtaining robust biological insights (Figure S1).

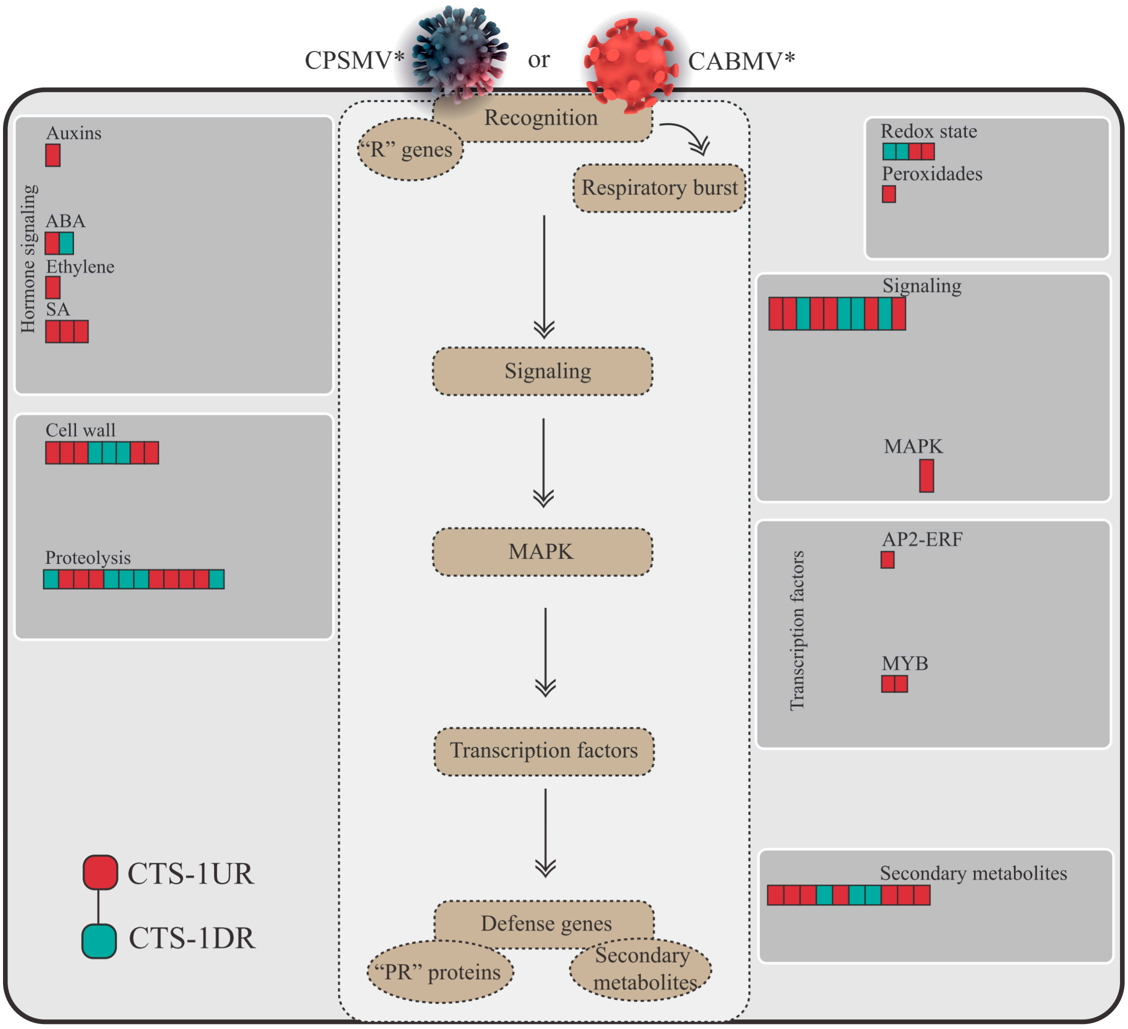

Figure S1. Transcripts from the CTS-16UR and CTS-16DR groups mapped onto the biotic stress response modules using the MapMan tool. Legend: *for illustrative purposes only; CTS-16UR set (made up by up-regulated transcripts in response to CABMV and CPSMV mechanical inoculations, at 16 hpi treatments); CTS-16DR set (made up by down-regulated transcripts in response to CABMV and CPSMV mechanical inoculations, at 16 hpi treatments); hpi (hours post inoculation); colored squares/rectangles indicate different transcripts
