# Supplementary figures and images for: Unlocking Cowpea’s Defense Responses: Conserved Transcriptional Signatures in the Battle against CABMV and CPSMV Viruses
Source: Life (Basel). 2023 Aug 15;13(8):1747. doi: 10.3390/life13081747 (PMC10455494; doi:10.3390/life13081747)

PLANT GROWTH

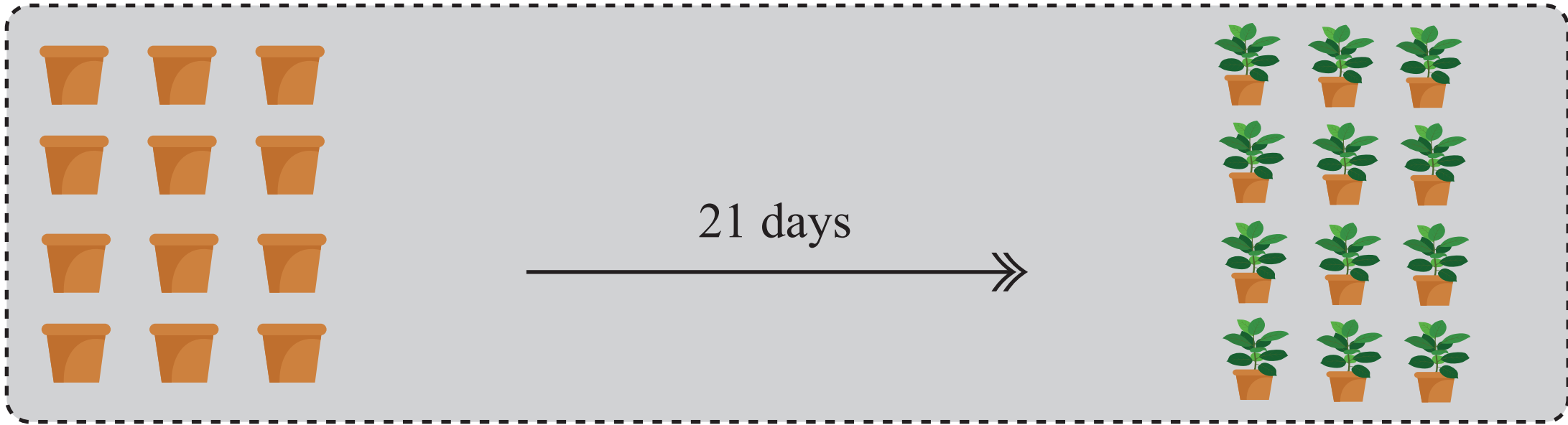

EXPERIMENTAL DESIGN

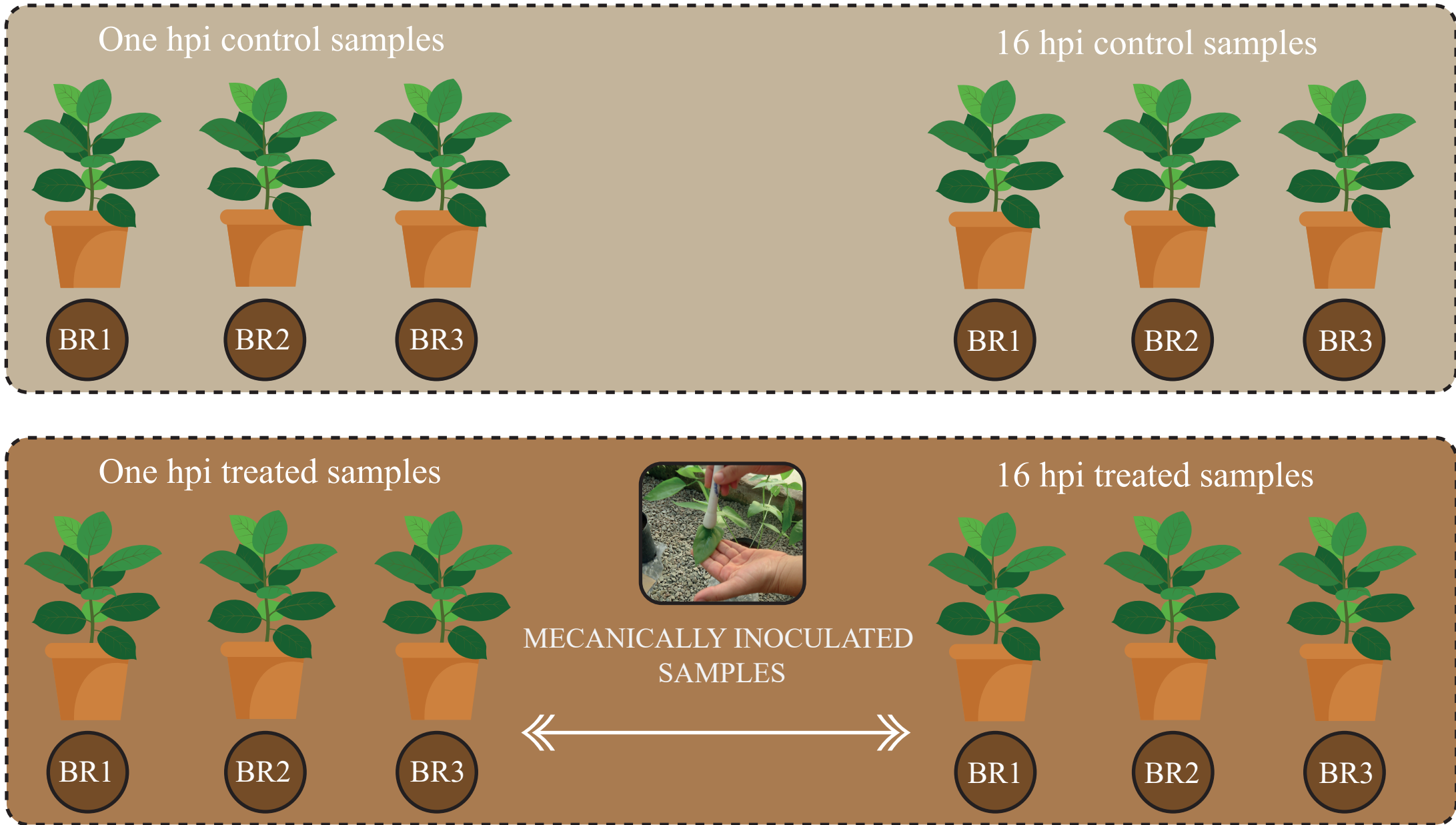

Supplement: Supplementary file 1 [file life-13-01747-s001.zip › Figure S1.pdf]
